# Unintended Consequences and Hidden Obstacles in Medicine Access in Sub-Saharan Africa

**DOI:** 10.3389/fpubh.2019.00342

**Published:** 2019-11-15

**Authors:** Iain Barton, Anton L. V. Avanceña, Nevashini Gounden, Ravi Anupindi

**Affiliations:** ^1^Imperial Logistics, Germiston, South Africa; ^2^Department of Health Management and Policy, School of Public Health, University of Michigan, Ann Arbor, MI, United States; ^3^Stephen M. Ross School of Business, University of Michigan, Ann Arbor, MI, United States

**Keywords:** pharmaceutical regulation, regulatory harmonization, drug access, Sub-Saharan Africa, drug quality, health impact

## Abstract

Many life-saving drugs are still inaccessible and unaffordable in low- and middle-income countries, particularly in Sub-Saharan Africa. This contributes to poor health outcomes, wider health and socioeconomic inequities, and higher patient spending on healthcare. While resource limitations facing national regulatory authorities (NRAs) contribute to the problem, we believe that (1) fragmented and complex drug regulations, (2) suboptimal enforcement of existing regulations, and (3) poorly designed disincentives for non-compliance play a larger role. These “unintended consequences” that are a direct result of our current regulatory regimes limit competition, keep drug costs high, and lead to shortages and the proliferation of illegitimate and unregistered drugs. While NRAs can gain a lot from increased investment in their work, regulatory harmonization and innovation can arrest and reverse the regulatory failures we still see today and improve medicine access in Africa. Unfortunately, harmonization initiatives in Sub-Saharan Africa have made modest impact and have done so slowly. We encourage greater attention and investment in harmonization and other downstream functions of NRAs. We also urge increased participation of national governments–particularly executive agencies in health and the treasury—and patient advocacy groups in advancing harmonization across the subcontinent.

## Introduction

**Figure 1:**
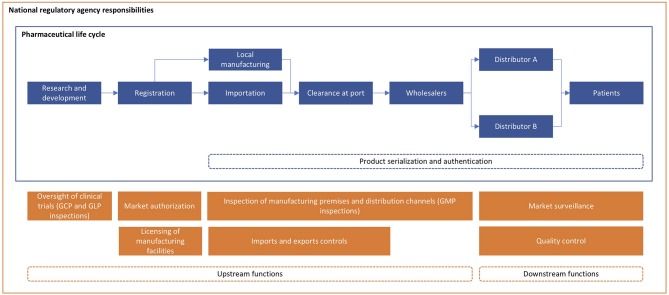
Roles and responsibilities of national regulatory agencies.

In most countries, a national regulatory agency (NRA) ensures that medicines are safe, effective, affordable, and are high-quality (1–4). NRAs develop and enforce pharmaceutical regulations that protect consumers who cannot judge on their own whether a drug is completely safe or authentic (4). Drug regulation also promotes health security and limits the rise of drug-resistant microbial diseases by ensuring that medicines have the correct and sufficient amount of active pharmaceutical ingredient. Many targets under Goal 3 of the Global Goals for Sustainable Development (e.g., achieving universal health coverage) can only be achieved when countries are supported by well-functioning and sufficiently- funded NRAs that safeguard access to essential medicines (2). Effective drug regulation can also build trust between the public and the government and promote research and development of new treatments (5). All in all, NRAs carry out a continuum of functions; upstream processes affect market entry and quality control, while downstream processes emphasize post-market surveillance (Figure 1).

Low- and middle-income countries (LMICs), particularly Sub-Saharan African (SSA) countries, have made great strides in establishing their own NRAs and drug regulatory regimes; however, the availability, affordability, and quality of life-saving drugs are still not guaranteed (6). In this perspective article, we propose a framework that explains how (1) fragmented and complex drug regulations, (2) suboptimal enforcement of regulations, and (3) poor disincentives for non-compliance create unintended consequences that lead to poor health outcomes and health inequities. We posit that in carrying out certain functions and advancing their legitimacy, NRAs often create a regulatory environment that discourages manufacturers from entering, or staying in, certain markets; this limits competition, keeps drug costs high, and leads to shortages and the proliferation of illegitimate and unregistered drugs. We argue that some of the issues around drug availability, affordability, and quality result not from poor NRA capacity (e.g., weak management structures, inadequate human, and financial resources) but from the misguided belief that each LMIC must establish its own NRA with its own standalone regulations. Finally, we urge the involvement of various stakeholders to effectively address these issues.

## Conceptual Framework

**Figure 2:**
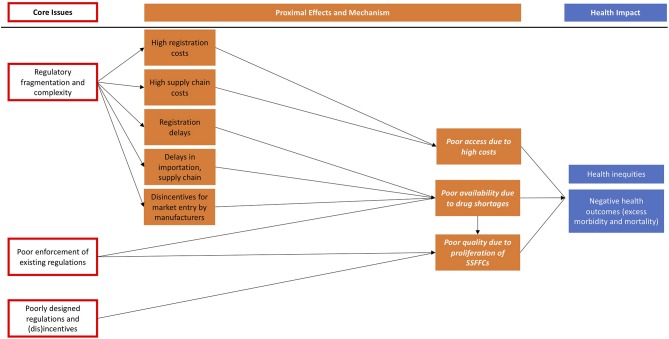
Framework for understanding the unintended consequences of regulatory fragmentation and complexity, poor enforcement, and ineffective disincentives.

We developed the conceptual framework (Figure 2) below based on a systematic review of peer-reviewed literature (in MEDLINE via PubMed), gray literature identified through snowball and convenience sampling, and insights from key informant interviews. We also drew from our experiences and insights as practitioners and researchers in supply chain management and organizers of one of the largest global health supply chain conferences that is now on its 12th year (7).

Our framework demonstrates how regulatory fragmentation, poor policy design, and enforcement issues lead directly or indirectly to unintended consequences, which in turn influence population health and health equity. It is important to note that other core issues such as corruption and complex international trade agreements are not depicted in the framework but are also important causes of poor affordability, availability, and quality of drugs (1, 8, 9). In the following section, we explain each pathway and provide evidence. We hope that this framework will be refined over time by empirical research.

### Regulatory Fragmentation and Complexity

Regulatory fragmentation and complexity arise from convoluted and duplicative requirements imposed on drug manufacturers by each NRA before granting initial market authorization (MA) or renewal. Examples include redundant and non-transparent registration procedures that are unaligned with international standards; widely divergent requirements across countries for establishing bioequivalence; complex country-specific labeling and packaging requirements; and costly requirements for good manufacturing practice (GMP) inspections (4, 10, 11). There may even be divergent rules between national and subnational regulations.

Once MA has been granted, fragmented and complex regulations can increase supply chain costs or lead to stockouts (Figure 2) (1). For example, regulations on residual shelf life of medicines at time of import, which were first imposed decades ago to protect against dumping of short-shelf life medicines, significantly reduce the ability of manufacturers to supply country orders from large batch runs or from more agile and responsive regional stock locations. These regulations also force manufacturers to supply using expensive modes (e.g., airfreight) to reduce delivery lead times, while others may even decide to withdraw from the market if operational costs are prohibitive relative to potential revenues.

The health impact of drug stockouts are significant, leading to delayed treatment initiation, incomplete treatments and treatment interruptions, disengagements with patients, and increased out-of-pocket expenditures (12–14). Consequently, patients face higher risks of developing drug-resistant or more severe forms of disease, complications, and death (12, 13, 15–17).

For new market entrants, fragmented, complex regulations can serve as a disincentive or barrier to supply (18). The “mosaic of regulations” that each country's NRA puts in place significantly delay registration or licensing (5). Work by Ahonkhai et al. (19) found that new, life-saving drugs will take 4–7 years to be approved in SSA after being submitted for approval to a well-resourced NRA such as the US Food and Drug Administration (FDA) or the European Medicines Agency (EMA) (19). These issues help explain why fewer medicines are available in SSA compared to other regions and countries, and why drug costs tend to remain higher for longer (20).

Fragmented, complex regulations can also lead to significant costs for a manufacturer. In a survey of 33 drug manufacturers operating in Africa, respondents cited high registration, retention, and GMP inspection costs as the main reason for deciding against entering certain African markets (11). Though published registration costs for new drugs are lower in SSA than in high-income countries (21), duplicative and resource-intensive regulatory processes require companies to invest significant financial resources to get a drug approved. The increased costs can be passed to consumers; some companies however, particularly manufacturers of generic medicines and other smaller-margin products, may decide against supplying the drug altogether. Thus, competition is limited, drug costs stay high, and health and social inequities are exacerbated as primarily well-off families can afford life-saving but expensive medications (1, 4, 5, 10).

When effective and legitimate drugs are inaccessible or unaffordable, demand is quickly filled by illegitimate drugs (4, 8). The World Health Organization (WHO) estimates that 1 in 10 medical products in LMICs are illegitimate, though this likely masks the wide heterogeneity across countries where over 35% of certain types of drugs (e.g., antimalarials) are substandard or falsified (2, 22, 23). A recent systematic review estimates that almost 19% of medicines in SSA and 13.7% in Asia are poor-quality (24).

Illegitimate and unregistered drugs can lead to poisoning, untreated disease or treatment failure, and premature death (4, 22, 25). A 2017 WHO report estimates that 72,000–169,000 deaths among children per year may be due to substandard and falsified antibiotics used to treat pneumonia, and an additional 116,000 (range: 64,000–158,000) caused by illegitimate antimalarials in SSA annually (26).

Illegitimate drugs also lead to significant opportunity costs for consumers and healthcare systems, as money, time, and efforts are wasted on ineffective medications and additional treatments (2, 4, 24, 27). Globally, the economic burden of illegitimate drugs is estimated to be between $10–200 billion (24). The same 2017 WHO report estimates the cost of additional care due to illegitimate antimalarials to be US$38.5 million per year (range: US$21.4 million−52.4 million) (26). In the Democratic Republic of Congo (DRC) alone, a recent modeling study estimates that illegitimate antimalarials cost $20.9 million (or 35% of malaria costs) in Kinshasa province and an additional $130 million in Katanga region (28).

As with stockouts, substandard and falsified drugs can also result in drug resistance, forcing additional costs in developing new drugs to replace ineffective regimens (2, 4, 23, 25, 29). For example, in the DRC development of antimalarial drug resistance is estimated to cost an additional $90 million per year in additional treatments (28). Finally, illegitimate drugs induce distrust among consumers, ultimately weakening other public health efforts (4, 26, 30).

### Poor Enforcement of Existing Regulations

Countries with stringent laws safeguarding pharmaceutical quality still experience many problems because regulations are poorly enforced, and illegitimate drugs still end up in ports and drug shops. News media have covered several raids and interceptions performed by local law enforcement in Africa (31–33). INTERPOL seized 420 tons of illegitimate drugs worth approximately US$21.8 million in West Africa during its Operation Heera in May–June 2017 (4, 34). Previous operations in East, Southern, and West Africa have also seized millions of units of illegitimate drugs (35).

The market for substandard and falsified drugs is substantial; the WHO estimates upwards of US$30 billion is spent on illegitimate drugs globally per year, while the Center for Medicines in the Public Interest thinks the amount is closer to US$75 billion (2, 9). The UN Office on Development and Crime believes the sale of illegitimate drugs in West Africa alone is worth more than the combined value of crude oil and cocaine trafficking (roughly US$1 billion) (4). Thus, as long as unscrupulous manufacturers have a good chance of getting away with their crime, illegitimate drugs are likely to persist and proliferate (4). Currently drug counterfeiters are less likely to be caught than narcotic drug traffickers, suggesting that significantly more effort can be directed toward stopping illegitimate medicines from spreading (9, 33).

Poor enforcement may be the result of resource constraints and poor capacity among NRAs (4). However, other factors such as low political commitment and prioritization, corruption, and ineffective management are also to blame (9). Poor hiring policies limit performance; for example, NRAs commonly hire pharmacists in roles requiring different competencies than what they were trained for. NRAs disproportionally allocate resources on upstream functions (Figure 1) that can be simplified or de-duplicated through regulatory harmonization (36). Finite NRA resources should instead be spent on downstream functions such as post-market surveillance and detection of illegitimate drugs.

### Poorly Designed Regulations and Disincentives

In many countries throughout SSA, disincentives for producing, selling, and distributing illegitimate drugs are insufficient to deter illegal manufacturers (37, 38). In fact, the penalties for drug falsifiers and counterfeiters are far more lenient than for drug traffickers who are often fined large sums and given long prison sentences when caught and successfully prosecuted (4). For example, the minimum prison sentence for a manufacturer of falsified drugs in Tanzania is 2 years, with a corresponding maximum civil penalty of up to US$57,000 (4). For drug traffickers, the minimum sentence is 30 years to a lifetime (39). Because counterfeiting and falsification are lucrative activities and are largely risk-free, the market for illegitimate drugs is believed to be larger than for illegal drugs (9). Given the health, social, and economic costs illegitimate drugs exact on LMICs, NRAs must push for legislation that increases the punishment for lawbreaking manufacturers. To be effective, penalties must meet or exceed the potential revenue from illegitimate drug production, trade, and sale. Policies should also address drug shortages and the high costs of genuine drugs as these can give rise to a significant unmet demand for cheap, accessible medicines—a void that illegitimate drugs are designed to fill (4, 9).

## What can be Done?

While NRAs can gain a lot from increased investment in their work, we think regulatory harmonization of current regulations and innovation can arrest and reverse the regulatory failures we still see today and improve medicine access in SSA.

### Defining Regulatory Harmonization and Its Benefits

Harmonization refers to the development and adoption of common technical requirements and guidelines for pharmaceutical regulation. Harmonization is not the same as convergence, which the FDA defines as the process in which “regulatory requirements across countries or regions become more similar or ‘aligned' over time.”(40). Harmonization goes further than convergence in that it emphasizes deduplication and mutual recognition of technical guidelines and standards, registration processes, and requirements across participating NRAs (40). Under convergence, countries may set similar but separate requirements for manufacturers applying for MA; with harmonization, several countries may participate in a joint assessment in lieu of country-by-country approval.

Harmonization will result in streamlined regulations and processes, which can reduce redundancy and drug registration times, leading to increased access to new and effective treatments (5, 23). For example, the Mexican NRA established a unilateral agreement with EMA that says the NRA will recognize findings from EMA's assessment of a drug manufacturer that is also applying for a new drug MA in Mexico (41). Mexico has similar arrangements with the US, Canada, Australia, and Switzerland.

SSA as a whole can benefit from similar mutual recognition agreements as they will reduce administrative and cost burdens faced by resource-limited NRAs and pharmaceutical manufacturers alike (11, 23). By relying on the expertise and resources of other countries, NRAs in SSA that are significantly more strapped for resources than Mexico's can focus their efforts and resources on downstream functions (Figure 1) (4, 6, 36, 42). Bilateral and multilateral donors should support NRAs' efforts to improve post-market surveillance, to streamline regulatory processes, and to enforce existing laws and regulations (17).

All-in-all, we believe harmonization will indeed strengthen resource-constrained NRAs in SSA and ensure the supply of effective and high-quality products. A 2018 modeling study by the global health organization PATH estimated that 23,000 lives in the East African Community (EAC) and in Zazibona countries (Botswana, Namibia, South Africa, Zambia, and Zimbabwe) could be saved by accelerating the authorization of dispersible amoxicillin tablets, an antibiotic used to treat childhood pneumonia, by 2 years (43).

### Current Harmonization Efforts

There are several regulatory harmonization efforts underway, though in practice many of them are focused only on convergence (Table A1 in Supplementary Material). Some initiatives claim to have demonstrated results, while others were recently established to overcome new challenges and persistent gaps; however, no impact has yet been demonstrated on the macro-scale issues described above such as the prevalence of illegitimate medicines and low registration rates due to regulatory cost and complexity. Most initiatives are also not legally binding, which can create issues with compliance and uniformity across countries. Finally, the participants of most initiatives are from the NRAs themselves, effectively excluding patient advocacy groups and government executives such as those from ministries of health or treasury.

Among the models of regulatory harmonization that exist, the EMA framework holds the most promise for the SSA region. The EMA's centralized procedure grants MA to a new drug that is valid for the 28 countries of the European Union and the three countries in the European Economic Area (44). The EMA assessment, which requires the approval of the European Commission, is usually completed within 210 days (45). The centralized procedure is required for many classes of drugs (e.g., HIV, cancer, and diabetes drugs) and optional for others. Drug manufacturers who wish to seek MA in one or several European countries can do so through national MA procedures or other routes (46). But because regulatory requirements and processes have been harmonized throughout the EU, data requirements and standards across MA routes are the same (4).

In Africa, the African Medicines Regulatory Harmonization (AMRH) initiative's regional project is leading efforts in pursuit of regulatory harmonization. Coordinated by the African Union's (AU) NEPAD Agency, the AMRH has led to improvements with real potential, including harmonization of (1) guidelines for registration of generic medicines, (2) GMP inspections, (3) quality management systems, and (4) information management systems in the EAC and the Southern African Development Community (SADC) (6, 17). The EAC has successfully conducted joint assessment of manufacturer dossiers, which has reduced drug approval timelines by 40–60% for branded medicines (6, 42, 47). As of 2017, 12 African countries have adapted the AU Model Law on Medical Products Regulation which aims to establish common pharmaceutical laws throughout the continent (6).

### A Path Forward: Some Recommendations

The progress of the AMRH should be supported and accelerated. To sustain the initiative's momentum, several actions should be undertaken. First, the joint assessment process can still benefit from further streamlining in the short term; for example, separate country registration forms and fees are still required in the EAC. In the long term, the AMRH should also move aggressively to establish the African Medicines Agency, which will serve as a pan-African regulatory agency (6). Second, the AMRH and NRAs should allocate resources on educating and encouraging manufacturers to use the regional MA route to expand access to drugs quickly and efficiently. Related to this, certain classes of drugs should be required to go through joint assessments as opposed to country-by-country MA. Third, the AMRH should swiftly replicate successes in the EAC and SADC regions to other economic communities in SSA. Throughout this process, we encourage ministers of health to be more involved and ensure political commitment at the national level. Fourth, the AMRH should work with the EMA, the FDA, and other NRAs in order to achieve optimal streamlining and de-duplication of efforts. It would be unfortunate to achieve sub-regional harmonization in the African continent yet preserve regulatory fragmentation and complexity at the continental level. To reduce barriers to medicines importation, trade authorities including ministries of commerce should also get involved. Finally, once the AMRH achieves regulatory harmonization, the initiative should continue its work streamlining and strengthening positive NRA functions such as post-market surveillance and enforcement.

In addition to regulatory harmonization, two innovations—serialization, which is the assignment of unique, traceable numbers to individual items, and mobile technology-based product verification—hold promise in fighting the scourge of illegitimate and unregistered drugs. Product verification through services like mPedigree and Sproxil allows consumers to determine whether drugs are authentic at the point of sale (48). Serialization using GS1 barcodes like 2D data matrices allow various agencies and actors along the supply chain to determine the source and authenticity of various drugs. Information from these technologies can then be used in cross-border intelligence and operations; for example, INTERPOL's collaborative operations have been successful in thwarting illegal drug manufacturers and distributions in select SSA countries and should be supported by manufacturers, governments, and donors (38).

## Conclusion

National regulatory authorities and pharmaceutical regulatory regimes are key to promoting access and quality of life-saving drugs. However, NRAs that have fragmented, unnecessarily complex, ineffective, and poorly enforced regulations contribute to poor health and health inequities. To date, NRA-led initiatives pursuing harmonization, while many, have delivered few results very slowly. We encourage NRAs, regional initiatives, national governments, and donors to exert significant leadership and action in pursuit of regulatory harmonization.

## Author Contributions

All authors listed have made a substantial, direct and intellectual contribution to the work, and approved it for publication.

### Conflict of Interest

IB is Healthcare Strategy Executive and NG is Healthcare Programme Manager at Imperial Logistics, a private logistics and supply chain management company that has commercial contracts with numerous pharmaceutical companies operating and trading in Africa, as well as a range of donors and national governments. The remaining authors declare that the research was conducted in the absence of any commercial or financial relationships that could be construed as a potential conflict of interest.
