# Supplementary material for: Unintended Consequences and Hidden Obstacles in Medicine Access in Sub-Saharan Africa
Source: Front Public Health. 2019 Nov 15;7:342. doi: 10.3389/fpubh.2019.00342 (PMC6873739; doi:10.3389/fpubh.2019.00342)
Supplement: Supplementary file 1 [file Table_1.DOC]

**Appendix Exhibit 1. Regulatory harmonization organizations and groups***

| **Name** | **Date established** | **Stated goal** | **Membership** | **Characteristics** | | | |
| --- | --- | --- | --- | --- | --- | --- | --- |
|  |  |  |  | *Legally binding* | *Knowledge-sharing and consensus-building* | *African country membership* | *Deduplication or mutual recognition an explicit goal* |
| **African Medicines Regulatory Harmonization (AMRH)** | 2009 | “The objective of the AMRH is to establish and improve standards and requirements related to the regulation of and access to safe, high-quality medicines for the African population.” | 55 member states of the AU | 🗸 | 🗸 | 🗸 | 🗸 |
| **Asia-Pacific Economic Cooperation (APEC) Harmonization Center** | November 2008 | “The APEC Harmonization Center's mission is to facilitate international cooperation for regulatory harmonization and trade facilitation of medical products.” | APEC members | x | 🗸 | x | x |
| **Association of Southeast Asian Nations (ASEAN) Consultative Committee on Standards and Quality (ACCSQ) Pharmaceutical Product Working Group (PPWG)** | 1999 | “The mission of the PPWG is to develop harmonisation schemes of pharmaceutical regulations of the ASEAN Member countries to complement and facilitate the objective of ASEAN Free Trade Area, particularly, the elimination of technical barriers to trade posed by regulations, without compromising on drug quality, efficacy, and safety.” | 10 member states of the ASEAN | 🗸 | 🗸 | x | 🗸 |
| **European Medicines Agency (EMA)** | 1995 | “EMA protects public and animal health in 28 EU Member States, as well as the countries of the European Economic Area, by ensuring that all medicines available on the EU market are safe, effective and of high quality.” | 28 member countries of the EU plus 3 EEA countries (Iceland, Liechtenstein, and Norway) | 🗸 | 🗸 | x | 🗸 |
| **Gul Central Committee for Drug Registration (GCC-DR)** | May 1999 | “The main role of the GCC-DR is to register pharmaceutical companies and their products through the joint coordination of scientific safety, efficacy, and quality of medicinal products.” | Bahrain, Kuwait, Oman, Qatar, Saudi Arabia, United Arab Emirates, and Yemen | x | 🗸 | x | 🗸 |
| **International Coalition of Medicines Regulatory Authorities (ICMRA)** | December 2013 | “ICMRA provides a global architecture to support enhanced communication, information sharing, crisis response and address regulatory science issues.” | 25 members, 5 associate members, 1 observer (WHO) | x | 🗸 | 🗸 | x^†^ |
| **International Conference of Drug Regulatory Authorities (ICDRA)** | 1980 | “The International Conference of Drug Regulatory Authorities (ICDRAs) provide drug regulatory authorities of WHO Member States with a forum to meet and discuss ways to strengthen collaboration.” | Open to all NRAs | x | 🗸 | x | x |
| **International Council for Harmonisation of Technical Requirements for Pharmaceuticals for Human Use (ICH)** | April 1990 | “ICH's mission is to achieve greater harmonisation worldwide to ensure that safe, effective, and high-quality medicines are developed and registered in the most resource-efficient manner.” | 15 members (7 countries, 6 industry representatives, and 2 standing regulatory members) and 24 observers | x | 🗸 | X^‡^ | 🗸 |
| **Pan American Network for Drug Regulatory Harmonization (PANDRH)** | November 1999 | “The PANDRH has the following objectives: strengthen the regulatory functions and systems of the countries of the Region; develop, approve and implement common proposals taking into account international guidelines and standards for regulatory convergence; and develop core competencies aimed at supporting and strengthening good regulatory practices and regulatory science in the Member States.” | PAHO member countries | x | 🗸 | x | x |
| **Pharmaceutical Inspection Co-operation Scheme (PIC/S)** | November 1995 | “Leading the international development, implementation and maintenance of harmonised GMP standards and quality systems of inspectorates in the field of medicinal products” | 52 (with additional 2 applicants and 3 pre-applicants) | x | 🗸 | 🗸 | 🗸 |

*AU African Union; EEA European Economic Area; EU European Union; GMP Good manufacturing practices; NRA National regulatory authority; PAHO Pan American Health Organization; WHO World Health Organization*

*^*^Not an exhaustive list*

*^†^Avoiding “duplication of activities among regulatory authorities” is a stated goal in the ICRMA strategic framework, but mutual recognition or streamlining not a priority*

*^‡^South Africa, the East African Community, and the Southern African Development Community, are observers but not full members*
